# Organ-Specific Transcriptome Analysis Identifies Candidate Genes Involved in the Stem Specialization of Bermudagrass (*Cynodon dactylon* L.)

**DOI:** 10.3389/fgene.2021.678673

**Published:** 2021-06-23

**Authors:** Si Chen, Xin Xu, Ziyan Ma, Jianxiu Liu, Bing Zhang

**Affiliations:** ^1^College of Animal Science and Technology, Yangzhou University, Yangzhou, China; ^2^Institute of Botany, Jiangsu Province and Chinese Academy of Sciences, Nanjing, China; ^3^Joint International Research Laboratory of Agriculture and Agri-Product Safety, Ministry of Education of China, Yangzhou University, Yangzhou, China

**Keywords:** *Cynodon dactylon*, organogenesis, transcriptomics, stolon, rhizome, starch

## Abstract

As an important warm-season turfgrass and forage grass species with wide applications, bermudagrass (*Cynodon dactylon* L.) simultaneously has shoot, stolon and rhizome, three types of stems with different physiological functions. To better understand how the three types of stems differentiate and specialize, we generated an organ-specific transcriptome dataset of bermudagrass encompassing 114,169 unigenes, among which 100,878 and 65,901 could be assigned to the Kyoto Encyclopedia of Genes and Genomes (KEGG) and the Gene Ontology (GO) terms, respectively. Using the dataset, we comprehensively analyzed the gene expression of different organs, especially the shoot, stolon and rhizome. The results indicated that six organs of bermudagrass all contained more than 52,000 significantly expressed unigenes, however, only 3,028 unigenes were enrich-expressed in different organs. Paired comparison analyses further indicated that 11,762 unigenes were differentially expressed in the three types of stems. Gene enrichment analysis revealed that 39 KEGG pathways were enriched with the differentially expressed unigenes (DEGs). Specifically, 401 DEGs were involved in plant hormone signal transduction, whereas 1,978 DEGs were transcription factors involved in gene expression regulation. Furthermore, in agreement with the starch content and starch synthase assay results, DEGs encoding starch synthesis-related enzymes all showed the highest expression level in the rhizome. These results not only provided new insights into the specialization of stems in bermudagrass but also made solid foundation for future gene functional studies in this important grass species and other stoloniferous/rhizomatous plants.

## Introduction

Bermudagrass (*Cynodon dactylon* L.) is an important warm-season turfgrass and forage grass species with wide applications. In warm regions around the world, different bermudagrass cultivars and their hybrid progenies with other *Cynodon* species are frequently used to generate high-quality turfs for multiple purposes (Reasor et al., 2016). With fast growth rate and special nutrition value, bermudagrass cultivars of forage type are highly valuable for cattle and dairy industry (Hill et al., 2001). In some countries, bermudagrass is also used as a traditional medical plant to cure several diseases, including anasarca, diarrhea and hemorrhage (Nagori and Solanki, 2011). On the other hand, bermudagrass is also a notorious weed in farmlands of warm regions, leading to a yield reduction of major crop plants (Jiménez-Brenes et al., 2019).

Unlike many well-known cereal grass species including rice, wheat, maize, and sorghum, bermudagrass has unique plant architectural characteristics that its stems are differentiated into shoot, stolon and rhizome (Dong and de Kroon, 1994). Among the three types of stems, shoot is an erect growing stem and widely seen in other plants, whereas stolon and rhizome are two kinds of specialized stems that grow horizontally aboveground and underground, respectively (Guo et al., 2020). With stolon and rhizome, bermudagrass can fast propagate by asexually clonal growth, which is exactly the reason why bermudagrass is both a useful turfgrass and a harmful weed (Horowitz, 1972; Fernandez, 2003). In the past several years, a few studies have successfully analyzed the functions of shoot, stolon and rhizome of bermudagrass and pointed out the difference among the three types of stems (Pornaro et al., 2019; Zhang et al., 2019). However, the molecular mechanism underlying the differentiation and development of the three stems in bermudagrass remains unclear.

Quantifying the differential expression of genes in various plant organs and tissues is vital to understand organogenesis and histogenesis of plants (Klepikova and Penin, 2019). In recent years, high-throughput comparative transcriptome analyses were successfully employed to identify candidate genes related to the differentiation and development of different organs and tissues in many plant species according to their temporal and spatial expression profiles. For example, a gene expression atlas encompassing 79 tissue samples was constructed to explore root development in maize (Stelpflug et al., 2016), whereas RNA sequencing of maize embryo, endosperm and seed resulted in the identification of novel transcripts possibly involved in embryo and seed development (Chen et al., 2014; Yi et al., 2019). Comparative transcriptome analysis of achenes and receptacles at four stages of fruit ripening revealed the possible important role of ethylene in receptacle ripening of strawberry (Sánchez-Sevilla et al., 2017). Transcriptome analysis of five organs identified putative 1,077 genes involved in rhizome development of *Miscanthus lutarioriparius* (Hu et al., 2017), whereas transcriptome analysis of the *Zingiber zerumbet* flower at two stages revealed 2,075 transcription factors (TFs) possibly involved in the flower development (Zhao et al., 2020). Transcriptome analyses were also conducted in bermudagrass to characterize the cold-resistance, salt-tolerance and other stress-responsive mechanisms (Chen L. et al., 2015; Hu et al., 2015; Melmaiee et al., 2015; Shi et al., 2015; Zhu et al., 2015; Fan et al., 2019). However, similar transcriptomics studies about the growth and development of bermudagrass are deficient.

In this study, we integrated the PacBio full-length transcriptome data and organ-specific Illumina sequencing results to characterize gene expression in the six organs of bermudagrass cultivar Yangjiang, especially the three types of stems: shoot, stolon and rhizome. The results not only provided new insights into the stem specialization of bermudagrass at the transcriptome level but also established a high-confidence database for future gene functional studies in this important grass species.

## Materials and Methods

### Plant Material

Bermudagrass (*C. dactylon*) cultivar Yangjiang was used in this study. The bermudagrass turf were grown in turfgrass plots of Yangzhou University (32°35′N, 119°40′E; 5 m a.s.l.; average annual temperatures: 22.4°C; average annual precipitation: 1,106 mm; annual average sunshine hours: 1,960 h; soil type: 80% river sand mixed with 20% peat soil) under routine management conditions (irrigation: as required to keep the soil moist; fertilization: four times/year; mowing: two times/month) for 2 years before the experiments were conducted. Approximately 15 g of leaves, shoots, stolons, rhizomes, roots and inflorescences were randomly collected from different plants (0.1 g per plant; 5 g sample collected from 50 plants as a replicate; three replicates) of the bermudagrass turf at flowering stages. The collected organ samples were frozen in liquid nitrogen and then stored at −80°C for RNA extraction, starch and soluble sugar content measurement, and enzyme activity assay.

### RNA Extraction, cDNA Library Construction, and Illumina Sequencing

Total RNA was extracted from 0.5 g of frozen organ samples using RNAprep pure Plant kit (Tiangen, Beijing, China). RNA integrity and concentration were determined by gel electrophoresis and Nanodrop 2000 spectrophotometer (Thermo Fisher Scientific, Wilmington, United States), respectively. Only samples of high-quality RNA (RNA integrity number ≥ 7) were used for following library preparation. cDNA libraries were prepared using Illumina TruSeq RNA Sample Preparation Kit (Illumina, San Diego, United States) according to the manufacturer’s instructions. Agilent 2100 Bioanalyzer (Agilent Technologies, Palo Alto, United States) was used to check the quality of the libraries. The qualified cDNA libraries were paired-end sequenced using Illumina HiSeq^TM^ 3000 (Illumina). For each organs, three sample replicates were sequenced to represent three biological replicates.

### Correction of the PacBio Sequencing Reads Using Illumina Sequencing Data

Adapter sequences, short reads (length <50 bp) and low quality reads (*Q*-value ≤ 30) were removed from the raw Illumina sequencing reads using Cutadapt software with default parameters (Martin, 2011). The obtained high-quality clean reads was used by LoRDEC software with default parameters (Salmela and Rivals, 2014) to construct de Bruijn graph and compared with the long consensus reads of the previously reported PacBio transcriptome of bermudagrass (Zhang et al., 2018). Corrected long consensus reads were further compared and clustered to non-redundant gene sequences, the unigenes, using the CD-HIT software with default parameters (-c 0.99 -aL 0.90 -AL 100 -aS 0.99 -AS 30) (Fu et al., 2012).

### Functional Annotation

The unigene sequences and their translated amino acid sequences were BLAST searched against the NCBI nucleotide (NT) database^1^, the NCBI non-redundant protein (NR) database (see text footnote 1), the Eukaryotic Ortholog Groups (KOG) database (see text footnote 1), the Gene Ontology (GO) database^2^, and the Kyoto Encyclopedia of Genes and Genomes (KEGG) pathway database^3^ with an *E*-value threshold ≤ 10^–5^ to obtain the annotation information.

### Gene Expression Analysis

The Illumina sequenced clean reads were mapped to the unigene sequences using Bowtie2 software of the RSEM package with default parameters (Li and Dewey, 2011). The numbers of mapped reads were converted to FPKM (fragments per kilobase of transcript per million mapped fragments) values. The log_2_ transformed FPKM values were applied to perform Hierarchical clustering using Pearson’s correlation distance in the Pvclust software package with default settings (Suzuki and Shimodaira, 2006). The significantly expressed unigenes were defined as FPKM value ≥ 1 (Nautiyal et al., 2020). The stably expressed unigenes were defined as the FPKM ratio (minimal versus maximal FPKM value in different organ samples) > 0.8 and the coefficient of variation (C.V., standard deviation divided by the average FPKM value) < 0.3 (Klepikova et al., 2016). The organ-enhanced unigenes were defined as FPKM value is five-fold above the average FPKM values of other organs, whereas organ-enriched unigenes were defined as FPKM value is five-fold above the FPKM values of any other organs (Uhlén et al., 2016). The DEGs were determined through comparison of FPKM values between two organs (three biological replicates) using DESeq2 software with the criterion of fold-change > 2.0 and *p*-value < 0.05 (Love et al., 2014).

### Pathway Enrichment Analysis and Transcription Factor Identification

The KEGG pathway enrichment analysis of DEGs was performed using KEGG Orthology Based Annotation System (KOBAS) web server with the significance cutoff of *p*-value < 0.05 (Xie et al., 2011). The TF families were identified through BLASTx search against known plant TFs recorded in PlantTFDB database^4^ with an *E*-value threshold ≤ 10^–5^.

### Quantitative PCR

cDNA was synthesized using the PrimeScript RT reagent kit (Takara, Dalian, China). RT-qPCR reactions were performed on a Mini Opticon Real-Time PCR System (Bio-Rad, Hercules, United States) using the SYBR Premix ExTaq (TaKaRa). PCR primer pairs were designed using the PrimerSelect software of the DNASTAR package (v7.1.0) and manually checked to ensure a high-efficient and accurate amplification (Supplementary Table 1). Relative gene expression level was quantified using the 2^–ΔΔCt^ method (Livak and Schmittgen, 2001).

### Soluble Sugar and Starch Content Determination

Soluble sugar and starch content were determined as previously described (Zhang et al., 2019). Briefly, frozen organ samples were baking dried to remove water and 0.1 g dry organ samples were ground to a fine powder using mortar and pestle. After washing with 100% acetone to remove interfering pigments, the powder was dissolved in 5 ml of 80% ethanol, incubated at 80°C in a water bath for 30 min and centrifuged at 8,000 × *g* for 10 min. For the soluble sugar content assay, the supernatants were mixed with a five-fold volume of 1% (m/v) anthrone dissolved in H_2_SO_4_. The mixture was held in a 100°C water bath for 10 min. The absorbance at 625 nm was determined using an Ultrospec 3300 Pro spectrophotometer (Amersham Biosciences, Uppsala, Sweden). The sugar content was calculated using the standard curve method. For the starch content assay, 3 ml of water was first added to redissolve the centrifuged pellet in a 100°C water bath for 10 min and then 2 ml of 1.1% (v/v) HCl was added to promote the degradation of starch to soluble sugar. After centrifugation at 8,000 × *g* for 10 min, the same procedures were performed to determine the sugar content of the supernatants, which represents the starch content.

### Starch Synthase Activity Assay

The starch synthase activity was assayed according to the previously described method with minor modifications (Zhou et al., 2018). Briefly, 0.5 g frozen organ samples were homogenized using mortar and pestle with 5 mL ice-cold HEPES-NaOH buffer (pH 7.5) containing 0.1% (w/v) PMSF. The homogenates were centrifuged at 5,000 × *g* for 15 min, and 100 μL supernatants were immediately transferred to 500 μL reaction solutions containing 50 mM HEPES-NaOH (pH 7.5), 1.6 mM adenosine diphosphate glucose, 15 mM DTT and 1 mg amylopectin. The reaction lasted for 20 min at 30°C and was stopped by boiling in a water bath for 2 min. After cooling, the produced ADP was converted to ATP by adding 100 μL of 40 mM phosphoenolpyruvate (PEP), 50 μL of 100 mM MgCl_2_ and 1 U pyruvate kinase (Sigma, Shanghai, China), followed by another 20 min incubation at 30°C. The produced ATP was determined using the ATP Assay System Bioluminescence Detection Kit (Promega, Madison, WI, United States) with a GloMax-Multi luminescence reader (Promega).

### Statistical Analyses

Unless otherwise specified, all the experiments were at least repeated for three biological and technical replicates. Tukey’s multiple comparison test was used for variation analyses among the different samples. The statistical analyses were performed with SPSS 16.0 statistical software package.

## Results

### Generation of a Bermudagrass Organ-Specific Transcriptome Dataset

**FIGURE 1:**
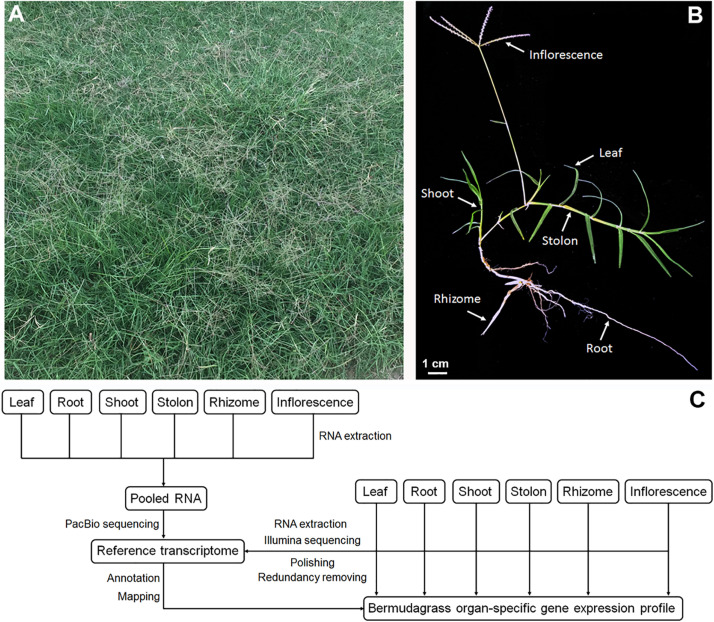
Experimental design for the bermudagrass organ-specific transcriptome dataset. **(A)** Photograph of the bermudagrass cultivar Yangjiang turf at flowering stage. **(B)** Photograph of the bermudagrass cultivar Yangjiang plant with six different organs. **(C)** Pipeline for the Illumina sequencing, reference transcriptome assembly and annotation, and gene expression profiling.

**FIGURE 2:**
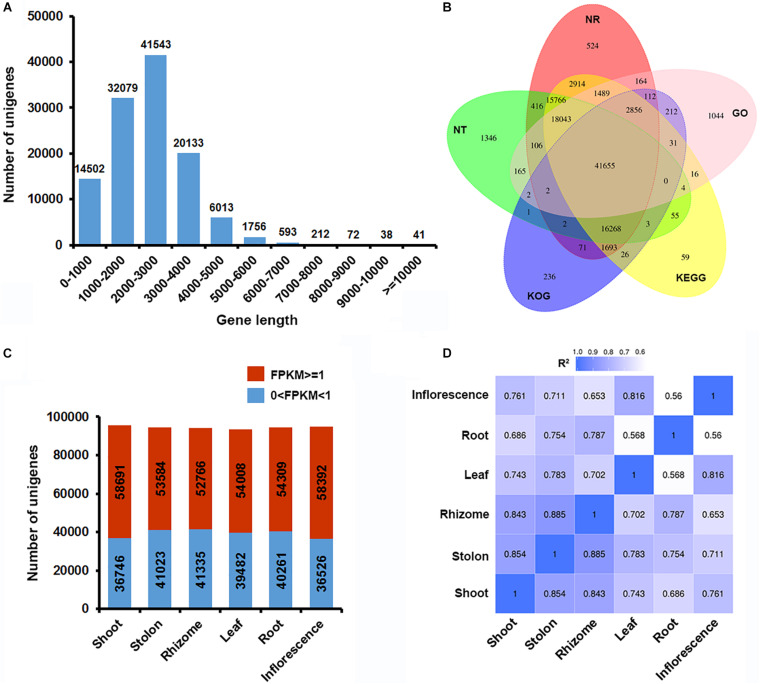
Overview of the bermudagrass organ-specific transcriptome dataset. **(A)** Unigene length distribution. **(B)** Venn diagram of NR, NT, GO, KOG, and KEGG annotation results of the bermudagrass reference transcriptome. **(C)** Distribution of significantly expressed unigenes (FKPM ≥ 1) in each organs. **(D)** Pearson’s correlation coefficients (*R*^2^) in pairwise comparisons of the bermudagrass organ-specific transcriptome dataset.

As a typical turf-type cultivar, bermudagrass cultivar Yangjiang forms uniform turfs with a high plant density (Figure 1A). Single mature bermudagrass plant is comprised of leaf, root, inflorescence, shoot, stolon, and rhizome (Figure 1B). In our previous study, we successfully used PacBio single-molecule long-read sequencing technology to obtain a full-length reference transcriptome of bermudagrass cultivar Yangjiang (Zhang et al., 2018). Here, we resampled the six organs of bermudagrass cultivar Yangjiang and sequenced the total mRNA of each organs using accurate Illumina sequencing technology. The short reads (average read length of 150 bp; average sequencing depth of 25.96) obtained from Illumina sequencing were compared with the long reads of PacBio sequencing to polish and correct the transcripts, which were further clustered together to remove redundant sequences (Figure 1C). After these steps, 114,169 unigenes with an average gene length of 2,237 bp were finally obtained. Notably, 82.12% of the unigenes (93,755) have a gene length between 1,000 and 4,000 bp (Figure 2A). Among the 114,169 unigenes, 92.37% (105,453) were successfully annotated by at least one database and 89.41% (102,081) could find homolog genes in NR database (Figure 2B and Supplementary Table 2). Notably, as many as 100,878 and 65,901 unigenes could be assigned to KEGG and GO terms, respectively.

Through mapping the Illumina short reads with the reference transcriptome and normalization through FPKM algorithm, we obtained the overall status of gene expression in the six organs (Supplementary Table 3). The six organs all had approximately 94,000 unigenes expressed with detectable reads (Figure 2C), however, the number of unigenes significantly expressed (FPKM ≥ 1) in at least one organ was only 71,653 (Supplementary Table 4). Notably, shoot and inflorescence both had more than 58,000 significantly expressed unigenes, whereas the other four organs only had about 52,000–54,000 significantly expressed unigenes (Figure 2C). Pearson correlation *R*^2^ values for all sequencing samples were between 0.82 and 1.0, with a mean value of 0.97, suggesting a high congruence of the biological replicates (Supplementary Figure 1). On the other hand, the expression profiles of different organs were highly divergent that the 1-R^2^ value were between 0.15 and 0.44, with a mean value of 0.25 (Figure 2D). Specifically, the *R*^2^ values of shoot, stolon and rhizome were similar and larger than that of other three organs, which is in accordance with their identities as different types of stems.

### RT-qPCR Validation of the Bermudagrass Organ-Specific Transcriptome Dataset

**FIGURE 3:**
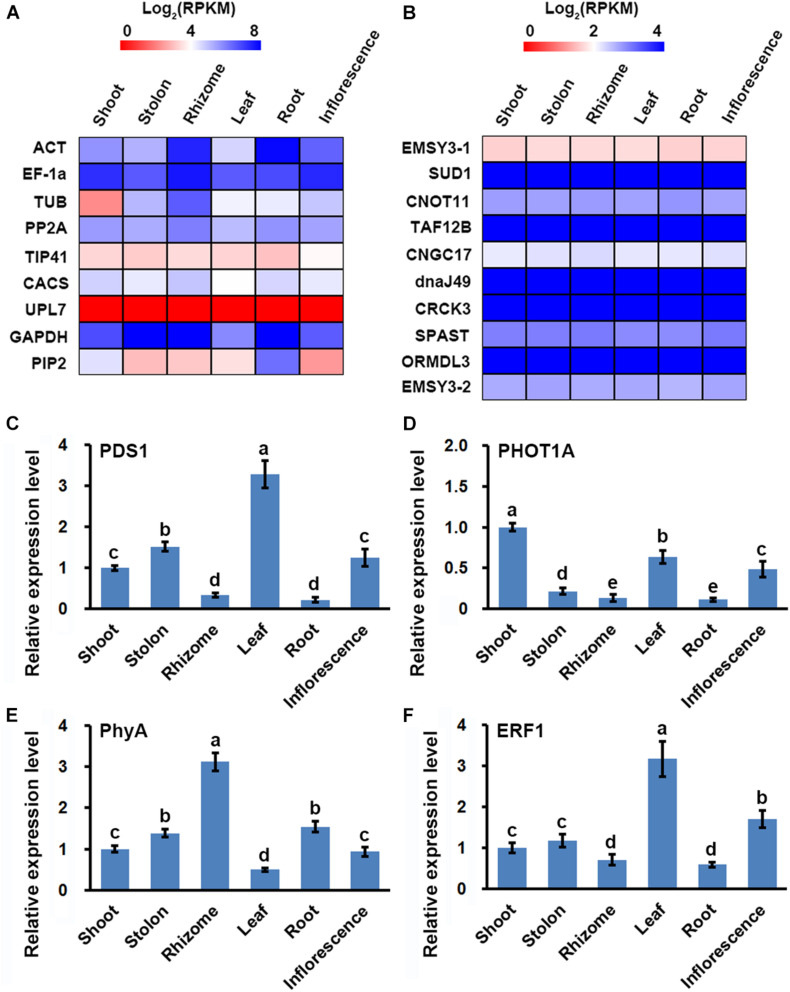
Identification of the stably expressed unigenes and its application in RT-qPCR assay of gene expression in six bermudagrass organs. Heatmap of the expression of **(A)** classical reference genes used for RT-qPCR analysis, and **(B)** stably expressed unigenes identified from the bermudagrass organ-specific transcriptome dataset. RT-qPCR analysis of the expression of **(C)** PDS1, **(D)** PHOT1A, **(E)** PhyA, and **(F)** ERF1 in six bermudagrass organs using the stably expressed SUD1 as a reference. Error bars represent SE. Different letters indicate significant differences determined by Tukey’s multiple comparison test.

RT-qPCR was routinely used to validate the reliability of transcriptome dataset (Ortiz-Ramírez et al., 2016), however, the reference gene for RT-qPCR must be correctly selected to obtain confident results (Liu et al., 2017). Using the organ-specific transcriptome dataset, we firstly evaluated the previously reported reference genes for quantitative analyses of gene expression in bermudagrass, including *alpha tubulin* (TUB), *actin* (ACT), *glyceraldehyde 3*-*phosphate dehydrogenase* (GAPDH), *elongation factor*-*1a* (EF1*α*), *TIP41*-*like family protein* (TIP41), *protein phosphatase 2A* (PP2A), *clathrin adaptor complex subunit* (CACS), and *E3 ubiquitin-protein ligase* (UPL7) (Chen Y. et al., 2015). We observed that the eight genes were not stably expressed in the six organs, showing divergent FPKM values in the organ-specific transcriptome dataset (Figure 3A and Supplementary Table 5). On the other hand, we found that ten unigenes, including *protein EMSY-LIKE 3 isoform X1* (EMSY3-1), *E3 ubiquitin ligase SUD1* (SUD1), *CCR4-NOT transcription complex subunit 11* (CNOT11), *transcription initiation factor TFIID subunit 12b* (TAF12B), *cyclic nucleotide-gated ion channel 17* (CNGC17), *chaperone protein dnaJ 49* (dnaJ49), *calmodulin-binding receptor-like cytoplasmic kinase 3* (CRCK3), *Spastin* (SPAST), *ORM1-like protein 3* (ORMDL3), and *protein EMSY-LIKE 3 isoform X2* (EMSY3-2), all showed relatively stable expression in the six organs with high ratios of minimal versus maximal expression level (>0.9) and small C.V. values (<0.1) (Figure 3B and Supplementary Table 6).

Using the stably expressed unigene SUD1 as a reference, we performed RT-qPCR assay of four randomly selected genes to test the validity of the organ-specific transcriptome dataset. The results indicated that *phytoene desaturase 1* (PDS1) and *ethylene responsive factor 1* (ERF1) genes were both highly expressed in leaf and weakly expressed in root, whereas *phototropin-1A* (PHOT1A) and *phytochrome A* (PhyA) were highly expressed in shoot and rhizome, respectively (Figures 3C–F). These results were highly correlated with the variations of FPKM values in the organ-specific transcriptome dataset since the Pearson correlation *R* value of the two results were all higher than 0.85 (Supplementary Table 7). Similar expression quantification result of ERF1 was also obtained in a study analyzing the function of ERF1 in cold tolerance of bermudagrass (Hu et al., 2020), which further verified the correctness of our results.

### Identification of Unigenes Differentially Expressed in Three Types of Stems of Bermudagrass

**FIGURE 4:**
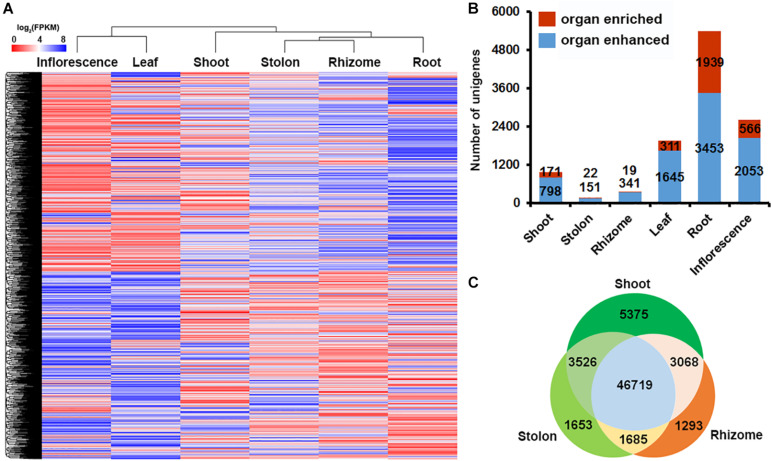
Gene expression profiling in six bermudagrass organs. **(A)** Hierarchical clustering of all the significantly expressed unigenes across six bermudagrass organs based on log_2_ transformed FPKM values. **(B)** Distribution of organ-enhanced and organ-enriched unigenes in each organs. **(C)** Venn diagram of the DEGs in pairwise comparisons of the three types of stems.

To obtain an overview of the transcriptional diversity of the six organs, we plotted the average FPKM value of the significantly expressed 71,653 unigenes (FPKM ≥ 1) in the six organs as a heatmap and analyzed the holistic expression pattern using hierarchical clustering. The results indicated that leaf and inflorescence were clustered in one group, whereas root, shoot, stolon, and rhizome were clustered in another group (Figure 4A). To further explore how different unigenes show an organ-specific expression profile, we analyzed the organ-enhanced and organ-enriched expression of the unigenes according to their FPKM values (Uhlén et al., 2016). The results indicated that there were 8,441 organ-enhanced unigenes and 3,028 organ-enriched unigenes in bermudagrass (Figure 4B and Supplementary Tables 8, 9). It’s noteworthy that root contained the largest proportion of enhance-expressed unigenes (40.90%) and enrich-expressed unigenes (64.04%), whereas unigenes enhance- or enrich-expressed in shoot, stolon and rhizome were much fewer. Specifically, only 22 and 19 unigenes were enrich-expressed in stolon and rhizome, respectively (Supplementary Table 9).

The small number of enhance- and enrich-expressed unigenes in shoot, stolon and rhizome compared with other organs suggested that stem specialization of bermudagrass is a complex process entailing the participation of multiple genes rather than a few unique genes. In order to identify unigenes possibly involved in stem specialization of bermudagrass, we further analyzed the gene expression profile of shoot, stolon and rhizome in a paired comparison manner and identified the differentially expressed unigenes among the three types of stems. The results indicated that 8,443 and 3,338 unigenes were differentially expressed between shoot and stolon, 5,179 and 4,361 unigenes were differentially expressed between stolon and rhizome, whereas 8,901 and 2,978 unigenes were differentially expressed between shoot and rhizome, respectively (Figure 4C). Furthermore, 5,375, 1,653, and 1,293 unigenes were preferentially expressed in shoot, stolon and rhizome, respectively (Figure 4C).

### Sucrose Metabolism Was Differentially Regulated in Three Types of Stems of Bermudagrass

**FIGURE 5:**
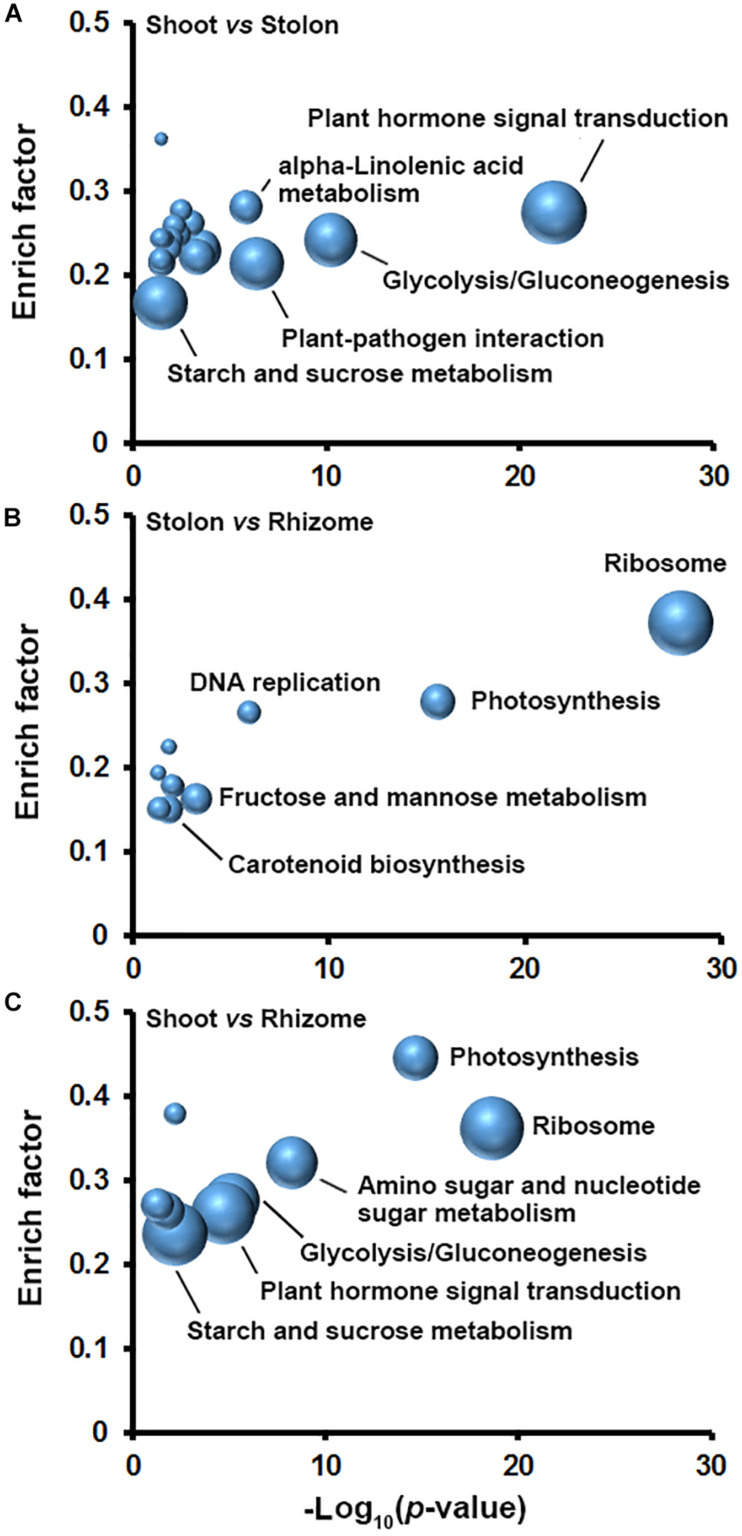
Pathways enriched with DEGs in three types of stems of bermudagrass. DEGs identified in pairwise comparisons of **(A)** shoot and stolon, **(B)** stolon and rhizome, and **(C)** shoot and rhizome were analyzed in KOBAS to obtain the biochemical pathways enriched with DEGs. Fold enrichment (*y* axis) is plotted as a function of statistical significance (minus log_10_ transformed *p*-value, *x* axis). Node size corresponds to the number of DEGs.

To better understand how the DEGs coordinate in the specialization of shoot, stolon, and rhizome in bermudagrass, KOBAS analyses were conducted to explore biological pathways enriched with DEGs in the three types of stems. The results indicated that as many as twenty pathways were significantly enriched with DEGs in shoot and stolon comparison and the top five pathways with largest number of DEGs were plant hormone signal transduction, glycolysis/gluconeogenesis, plant-pathogen interaction, starch and sucrose metabolism, and alpha-linolenic acid metabolism (Figure 5A). In stolon and rhizome comparison, there were ten pathways significantly enriched with DEGs, including ribosome, photosynthesis, fructose and mannose metabolism, carotenoid biosynthesis, and DNA replication (Figure 5B). In shoot and rhizome comparison, nine pathways were significantly enriched with DEGs and the top five pathways were plant hormone signal transduction, ribosome, starch and sucrose metabolism, glycolysis/gluconeogenesis, and photosynthesis (Figure 5C).

**FIGURE 6:**
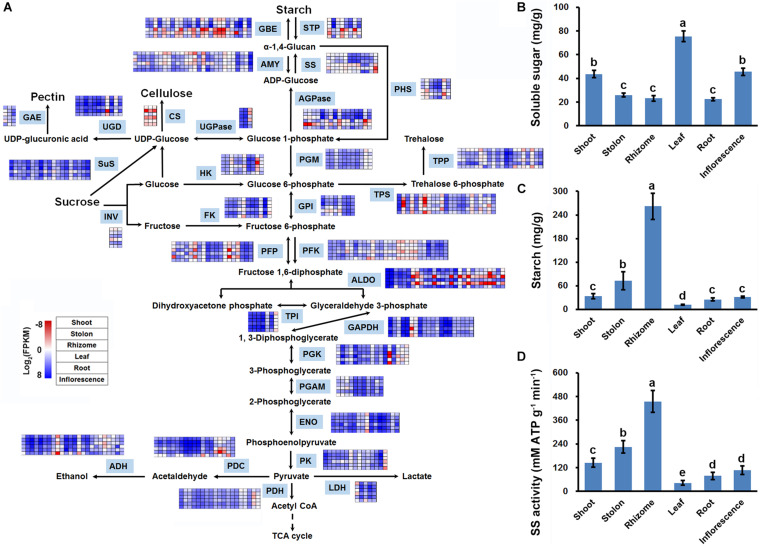
Sucrose metabolic activity analysis in three types of stems of bermudagrass. **(A)** DEGs identified in pairwise comparisons of three stems were shown in the sucrose metabolic reactions. **(B)** Soluble sugar content, **(C)** Starch content, and **(D)** Activities of starch synthase in the six organs of bermudagrass. Error bars represent SE. Different letters indicate significant differences determined by Tukey’s multiple comparison test.

In plants, sucrose is degraded by glycolysis to provide energy and carbon skeleton for amino acid and other secondary metabolite biosynthesis, and also can be used for starch synthesis (Ruan, 2014). KOBAS analysis results indicated that the two routes of sucrose metabolism were both significantly enriched with DEGs in the three stems (Figure 5). Functional annotation indicated that there are 449 DEGs encoding different isoforms of 41 enzymes to catalyze multiple reactions of glycolysis/gluconeogenesis, and starch and sucrose metabolism (Supplementary Table 10). Interestingly, expression profiling indicated that 55 DEGs encoding starch synthesis-related enzymes, including glucose-1-phosphate adenylyltransferase (AGPase), starch synthase (SS), and starch branching enzyme (GBE), all showed relatively higher expression level in stolon and rhizome (Figure 6A). Furthermore, DEGs encoding SS and GBE all showed the highest expression level in the rhizome. By contrast, enzymes catalyzing the degradation of sucrose by glycolysis, especially fructose-bisphosphate aldolase (ADO), showed relatively higher mRNA expression level in the shoot and the lowest expression level in the rhizome (Figure 6A). In agreement with the transcriptomics analysis results, soluble sugar measurement results revealed that leaf has the highest soluble sugar content (>70 mg/g), shoot and inflorescence both have a medium soluble sugar content (about 40 mg/g), whereas stolon, rhizome and root all showed the lowest soluble sugar content (<30 mg/g) (Figure 6B). In contrast, starch measurement results indicated that rhizome has the highest starch content (>200 mg/g). Stolon also accumulate substantial starch (about 80 mg/g), whereas other four organs all showed relatively low starch content (<40 mg/g) (Figure 6C). Furthermore, enzyme activity assay indicated that rhizome and leaf has the highest and lowest SS enzyme activity, respectively (Figure 6D). These results collectively suggested that sucrose assimilated in the leaf by photosynthesis was mainly transported to the rhizome and stored in the form of starch.

### Phytohormone and Transcription Regulatory Networks in Three Types of Stems of Bermudagrass

**FIGURE 7:**
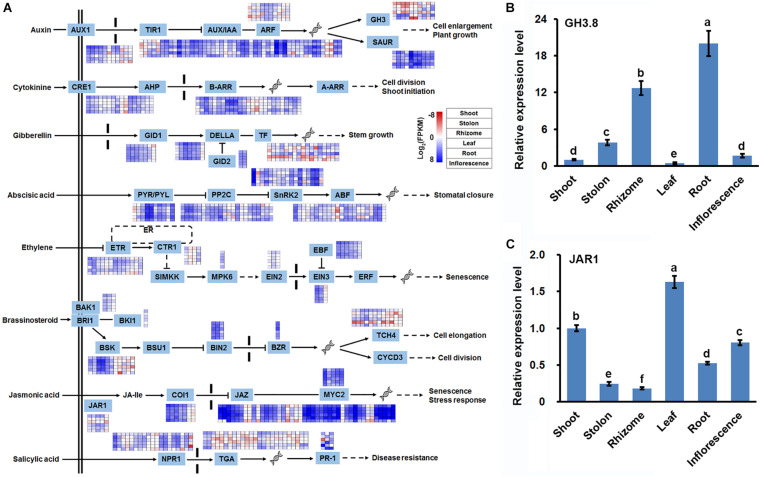
Expression profile of unigenes involved in plant hormone signal transduction. **(A)** DEGs identified in pairwise comparisons of three stems were shown in the eight phytohormone signaling transduction pathways. RT-qPCR analysis of the expression of **(B)** GH3.8, and **(C)** JAR1 in six bermudagrass organs using the stably expressed SUD1 as a reference. Error bars represent SE. Different letters indicate significant differences determined by Tukey’s multiple comparison test.

Considering that plant hormone signal transduction were also significantly enriched with DEGs in three types of stems (Figure 5), in combination with the important regulatory roles of phytohormone in stem growth and development (Gamuyao et al., 2017; Ge et al., 2019), we thoroughly analyzed the expression profile of 401 DEGs participating in plant hormone signal transduction. Among the 401 DEGs, 89, 41, 33, 71, 30, 31, 59, and 47 DEGs were annotated as auxin, cytokinine, gibberellin, abscisic acid, ethylene, brassinosteroid, jasmonic acid (JA), and salicylic acid signal transduction-related genes, respectively. Furthermore, the 401 DEGs were distributed in 35 of the 42 gene families constituting the different plant hormone signal transduction pathways (Figure 7A and Supplementary Table 11). Interestingly, expression profiling of the 35 gene families indicated that DEGs belonging to Gretchen Hagen 3 (GH3) family all showed relatively lower expression levels in shoot and stolon, whereas DEGs assigned as jasmonate resistant 1 (JAR1) all showed highest expression levels in the shoot and lowest expression levels in the rhizome (Figure 7A). RT-qPCR analyses of two unigenes, i2_LQ_mixture_c21936/f1p17/2403 and i2_LQ_mixture_c18254/f1p2/2233, which were annotated as *GH3.8* and *JAR1*, respectively, obtained similar results (Figures 7B,C). These results strongly suggested that auxin and JA signals were differently responded in three types of stems of bermudagrass.

**FIGURE 8:**
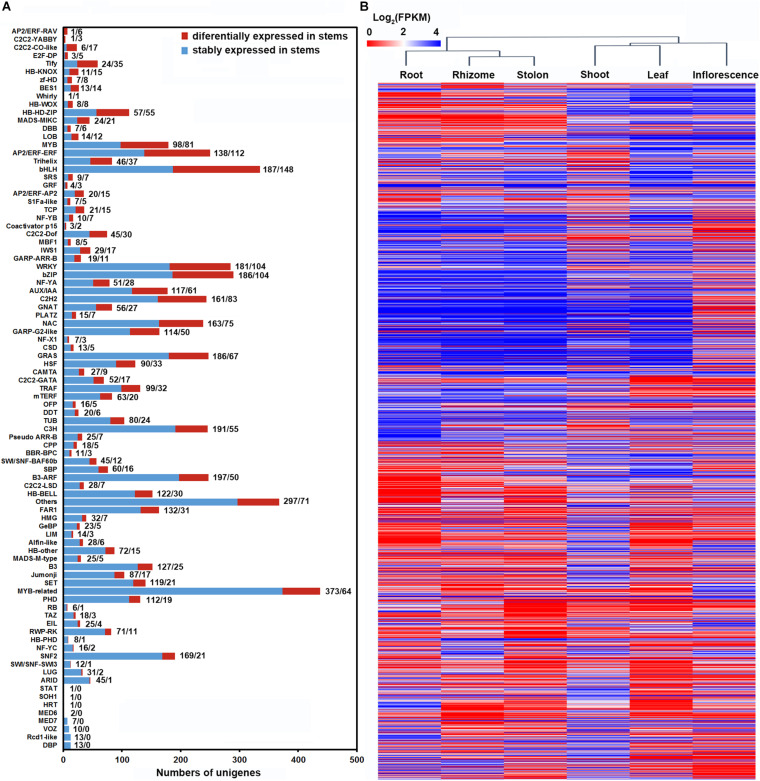
Differential expression of TFs in three types of stems of bermudagrass. **(A)** Distribution of TFs differentially expressed in three types of stems across each TF families. **(B)** Hierarchical clustering of the differentially expressed TFs across six bermudagrass organs based on log_2_ transformed FPKM values.

Transcription factors play essential regulatory roles in plant organ differentiation and development (Durgaprasad et al., 2019; Soyano et al., 2019). To provide insights into the transcription regulatory mechanism underlying shoot, stolon and rhizome specialization in bermudagrass, we further analyzed the dynamic expression of TFs in the three types of stems. Totally, 1,978 TFs belonging to 81 different families were found to be differentially expressed in the three types of stems, whereas 5,099 TFs were not differentially expressed (Figure 8A and Supplementary Table 12). Among the 81 TF families, bHLH, AP2/ERF, bZIP, WRKY, C2H2, MYB, and NAC all contained more than 70 DEGs, implying their in-depth participation in the shaping of the three stems. Notably, many TF families, including ARF, TIFY, were also classified as plant hormone signal transduction gene families (Figure 7A and Supplementary Table 11). Lastly, we also analyzed the expression pattern of the 1,978 TFs using hierarchical clustering. The results indicated that root, rhizome and stolon were clustered in one group, whereas shoot, leaf and inflorescence were clustered in another group (Figure 8B).

## Discussion

PacBio sequencing technique is an efficient approach to sequence long DNA molecules, leading to its widely application in full-length reference transcriptome construction of many plant species (Teng et al., 2019; Zhou et al., 2019; Xie et al., 2020). However, due to the inherent high error rate, transcriptome analyses based on PacBio sequencing is error prone and requires adjustment (Mahmoud et al., 2019). In this study, we successfully used the accurate Illumina sequencing data of six bermudagrass organs to correct the reference transcriptome obtained by PacBio sequencing (Zhang et al., 2018). After polishing and correction, the unigene number of the bermudagrass reference transcriptome was increased from 78,192 to 114,169, whereas the average unigene length was decreased from 2,317 bp to 2,237 bp (Figure 2A). The more accurate transcript information provided solid foundation for future molecular and breeding studies of this important grass species.

Identification of genes stably expressed in different tissues, organs, and growth conditions is important for the normalization of gene expression among samples in gene functional studies (Liu et al., 2017). In this study, we found that eight classical reference genes showed divergent expression pattern in different organs, whereas other ten unigenes were stably expressed in the six organs (Figure 3). Interestingly, although the ten unigenes participated in some essential cellular processes, including regulation of chromatin states (EMSY3-1 and EMSY3-2), protein degradation (SUD1), sphingolipid synthesis (ORMDL3), and transcription regulation (CNOT11 and TAF12B), they were not classical housekeeping genes (Robles et al., 2007; Breslow et al., 2010; Tsuchiya and Eulgem, 2011; Doblas et al., 2013; Laribee et al., 2015). These results were in line with the findings of transcriptomics survey of reference genes in other plants, which all identified many novel stably expressed genes (Mu et al., 2019; Wang et al., 2019; Long et al., 2020).

Using the organ-specific transcriptome dataset, we successfully analyzed the holistic gene expression of different organs in bermudagrass. Clustering analysis indicated that leaf and inflorescence showed similar gene expression profiles, whereas gene expression profiles in root, shoot, stolon, and rhizome are highly similar (Figure 4A). It was widely recognized that flowers are attractive and reproductive sexual leaf-like organs (Ruelens et al., 2017), thus it’s not surprising that leaf and inflorescence of bermudagrass have similar gene expression profiles. The clustering of root with the three types of stems in another group is also understandable because root and stem have similar vascular tissue composition and both function in solute transport and mechanical support (Ruonala et al., 2017). Interestingly, clustering analysis of TFs in the six organs obtained a different result that root, rhizome and stolon were clustered in one group, whereas shoot, leaf and inflorescence were clustered in another group (Figure 8B). Considering that shoot, leaf and inflorescence are three aboveground organs growing in the sunshine, whereas root, rhizome and stolon are grown underground or shaded by the canopy, this result implied that light might be an important factor to induce the differential expression of TFs in these organs (Xie et al., 2016; Anna et al., 2019).

Our study also provided many new insights into the specialization of stems in bermudagrass. Firstly, gene expression profiling, sugar and starch content assay as well as SS enzyme activity analysis strongly suggested that sucrose was differently metabolized in the three types of stems (Figure 6). In shoot, sucrose was mainly degraded through glycolysis, whereas in stolon and rhizome, especially rhizome, sucrose was efficiently transformed to starch. This result was in line with the previous suspicions that rhizome of bermudagrass functions as a storage organ (Dong and de Kroon, 1994; Pornaro et al., 2019) and shoot has relatively higher glycolysis activity (Zhang et al., 2019). Secondly, expression profiling and RT-qPCR both indicated that *GH3* and *JAR1* were preferentially expressed in rhizome and shoot, respectively (Figure 7). *GH3* encodes an auxin-amido synthetase protein to promote the inactivation of auxin, thereby inhibiting the auxin signaling transduction (Aoi et al., 2020). On the other hand, *JAR1* encodes a JA-conjugating enzyme to catalyze the transformation of JA to active JA-isolecucine (Chen et al., 2018). The synergistic regulation of *GH3* and *JAR1* expression could lead to variant auxin and JA contents in different types of stems to promote their morphogenesis and functional specialization.

## Conclusion

In summary, an organ-specific transcriptome dataset of bermudagrass cultivar Yangjiang was successfully constructed in the current study. Comprehensive gene expression in six organs of bermudagrass, including leaf, root, inflorescence, shoot, stolon and rhizome, were analyzed using the transcriptome dataset. Gene expression profiling indicated that the expression of 8,441 and 3,028 unigenes were enhanced and enriched in different organs, respectively. Paired comparison further revealed that totally 11,762 unigenes were differentially expressed among shoot, stolon and rhizome. Notably, 401 unigenes participating in plant hormone signal transduction, 449 unigenes encoding enzymes involved in glycolysis/gluconeogenesis and starch/sucrose metabolism, and 1,978 TFs belonging to 81 families were all identified to be differentially expressed in the three types of stems, implying the involvement of phytohormone, sucrose metabolism and TFs in the specialization of stem organs in bermudagrass. These results provided essential information for future functional studies of specific genes regulating the growth and development of different stems in bermudagrass and other plants.

## Data Availability Statement

RNA-seq data generated in this work has been submitted to the Bioproject database in NCBI under the accession number PRJNA685207.

## Author Contributions

BZ conceived and designed the study. SC, XX, and ZM conducted the experiments and analyzed the data. BZ and JL wrote the manuscript. All authors read and approved the manuscript.

## Conflict of Interest

The authors declare that the research was conducted in the absence of any commercial or financial relationships that could be construed as a potential conflict of interest.

## Footnotes

1 http://www.ncbi.nlm.nih.gov

2 http://www.geneontology.org

3 http://www.genome.jp/kegg

4 http://planttfdb.gao-lab.org/

## Supplementary Material

Supplementary Figure 1 Pearson’s correlations for all the sequencing samples.

Supplementary Table 1 Primers used for RT-qPCR.

Supplementary Table 2 Annotation information of the unigenes in NR database.

Supplementary Table 3 Sequencing and mapping statistics for each organ samples.

Supplementary Table 4 Expression profiles of the significantly expressed unigenes.

Supplementary Table 5 Expression profiles of the unigenes previously used as reference genes in RT-qPCR.

Supplementary Table 6 Unigenes stably expressed in different organs.

Supplementary Table 7 Expression profiles of four selected unigenes in different organs.

Supplementary Table 8 Unigenes with enhanced expression in specific organs.

Supplementary Table 9 Unigenes with enriched expression in specific organs.

Supplementary Table 10 Expression profiles of the unigenes involved in sucrose metabolism and differentially expressed in three types of stems.

Supplementary Table 11 Expression profiles of the unigenes involved in plant hormone signal transduction and differentially expressed in three types of stems.

Supplementary Table 12 Expression profiles of the transcription factors possibly involved in stem specialization.
