# Supplementary figures and images for: Organ-Specific Transcriptome Analysis Identifies Candidate Genes Involved in the Stem Specialization of Bermudagrass (Cynodon dactylon L.)
Source: Front Genet. 2021 Jun 23;12:678673. doi: 10.3389/fgene.2021.678673 (PMC8260954; doi:10.3389/fgene.2021.678673)

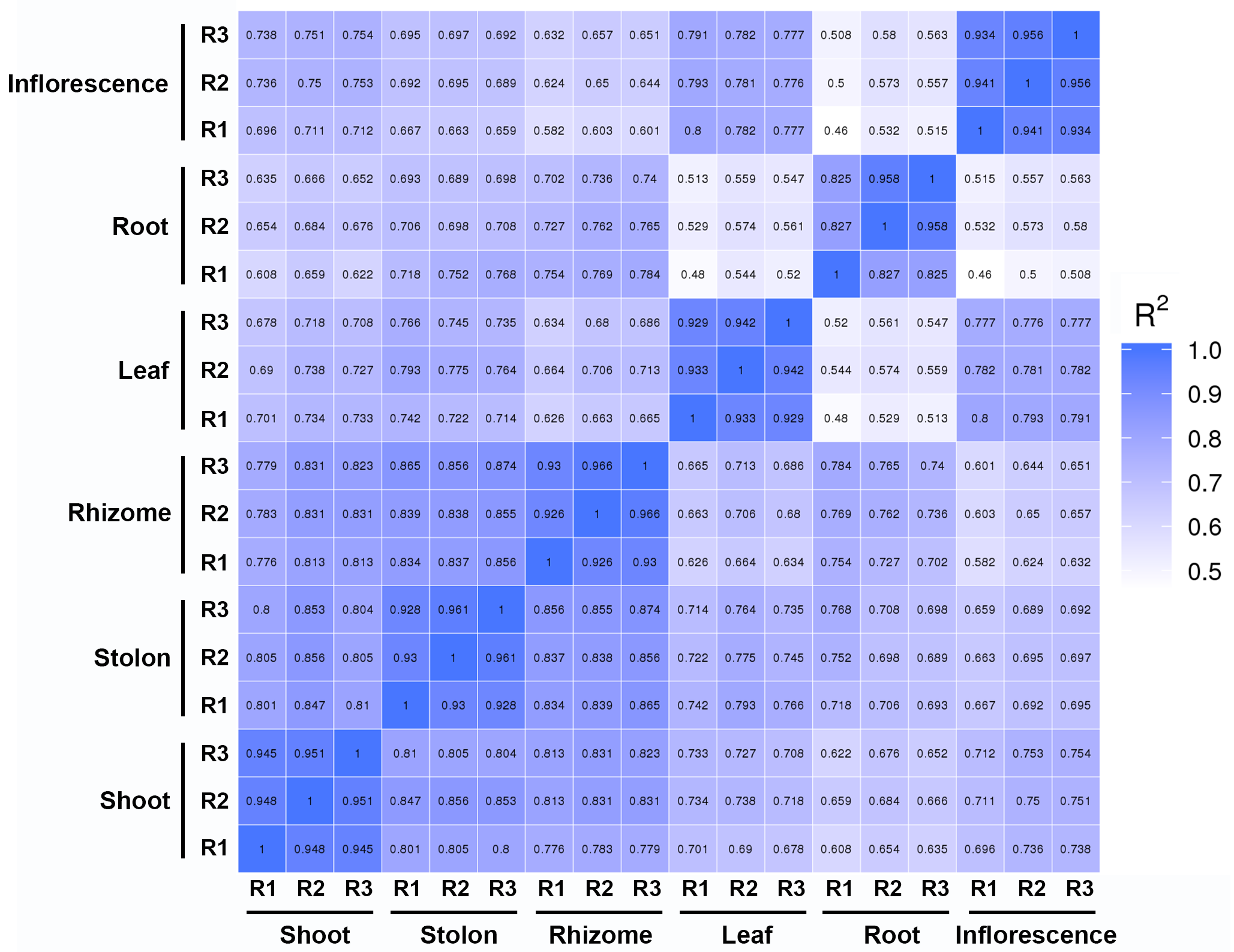

Supplement: Supplementary Figure 1 — Pearson’s correlations for all the sequencing samples. [file Image_1.TIF]
